# Risk and consequences of chemotherapy-induced neutropenic complications in patients receiving daily filgrastim: the importance of duration of prophylaxis

**DOI:** 10.1186/1472-6963-14-189

**Published:** 2014-04-27

**Authors:** Derek Weycker, Rich Barron, John Edelsberg, Alex Kartashov, Jason Legg, Andrew G Glass

**Affiliations:** 1 Policy Analysis Inc. (PAI), Four Davis Court, Brookline, MA, USA; 2 Amgen Incorporation, Thousand Oaks, CA, USA; 3 Center for Health Research, Kaiser Permanente Northwest, Portland, OR, USA

**Keywords:** Filgrastim, Granulocyte colony-stimulating factor, Febrile neutropenia, Cost, Neoplasms

## Abstract

**Background:**

To examine duration of daily filgrastim prophylaxis, and risk and consequences of chemotherapy-induced neutropenic complications (CINC) requiring inpatient care.

**Methods:**

Using a retrospective cohort design and US healthcare claims data (2001–2010), we identified all cancer patients who initiated ≥1 course of myelosuppressive chemotherapy and received daily filgrastim prophylactically in ≥1 cycle. Cycles with daily filgrastim prophylaxis were pooled for analyses. CINC was identified based on hospital admissions with a diagnosis of neutropenia, fever, or infection; consequences were characterized in terms of hospital mortality, hospital length of stay (LOS), and CINC-related healthcare expenditures.

**Results:**

Risk of CINC requiring inpatient care–adjusted for patient characteristics–was 2.4 (95% CI: 1.6-3.4) and 1.9 (1.3-2.8) times higher with 1–3 (N = 8371) and 4–6 (N = 3691) days of filgrastim prophylaxis, respectively, versus ≥7 days (N = 2226). Among subjects who developed CINC, consequences with 1–3 and 4–6 (vs. ≥7) days of filgrastim prophylaxis were: mortality (8.4% [n/N = 10/119] and 4.0% [3/75] vs. 0% [0/34]); LOS (means: 7.4 [N = 243] and 7.1 [N = 99] vs. 6.5 [N = 40]); and expenditures (means: $18,912 [N = 225] and $14,907 [N = 94] vs. $13,165 [N = 39]).

**Conclusions:**

In this retrospective evaluation, shorter courses of daily filgrastim prophylaxis were found to be associated with an increased risk of CINC as well as poorer outcomes among those developing this condition. Because of the limitations inherent in healthcare claims databases specifically and retrospective evaluations generally, additional research addressing these limitations is needed to confirm the findings of this study.

## Background

Neutropenia is a common side effect of myelosuppressive chemotherapy that both increases the risk of infection and diminishes patients’ ability to fight infection. When neutropenic patients become febrile, the high likelihood of infection and serious consequences thereof usually results in hospitalization [1,2]. FN, as well as severe or prolonged neutropenia, also can interfere with the planned delivery of treatment and adversely affect important patient outcomes [2-11].

Clinical practice guidelines recommend primary prophylactic use of a colony-stimulating factor (CSF)–which has been shown to reduce the risk of FN in clinical trials–when the risk of FN is 20% or higher [2]. While the American Society of Clinical Oncology (ASCO) initially recommended that CSF prophylaxis be administered only when FN risk is 40% or higher, in 2006, ASCO lowered the threshold to 20% based on data highlighting the importance of FN-related hospitalization as an outcome and evidence demonstrating the value of CSF prophylaxis in reducing the risk of FN, the risk of FN-related hospitalization, and the associated use of IV anti-infective agents [2,12,13]. The CSF filgrastim, which is widely used in clinical practice as prophylaxis against FN, requires daily administration during each cycle until neutrophil recovery occurs (in clinical trials, given typically for 10–11 days [and up to 14 days] until absolute neutrophil count [ANC] ≥10 × 10^9^/L) [14-19].

In clinical practice, patients often receive shorter courses of daily filgrastim prophylaxis than those administered to subjects in the clinical trial setting [14-16,20-24]. While published studies suggest that shorter courses of daily filgrastim prophylaxis are associated with an increased risk of hospitalization for CINC, these studies focused on selected tumor types or employed data that now are over a decade old [23,24]. During the past decade, use of chemotherapy and supportive care in clinical practice–as well as recommended use of these agents in authoritative guidelines–has changed considerably [2,25-27], Moreover, only one study has examined whether CSFs may favorably impact clinical outcomes and economic costs when CINC develop despite CSF prophylaxis, and it was published a decade ago and focused on elderly patients with a single type of cancer (non-Hodgkin’s lymphoma) [28]. We therefore undertook a new study to evaluate the relationship between the duration of daily filgrastim prophylaxis, and the risk and consequences of CINC requiring inpatient care using a large healthcare claims database. While such databases often lack detailed clinical information (e.g., on absolute neutrophil counts), they offer access to information on the health profile and healthcare utilization of tens of millions of covered lives.

## Methods

### Data source

Patient-level information from two large healthcare claims databases–the Thomson Reuters MarketScan Commercial Claims and Encounters and Medicare Supplemental and Coordination of Benefits Database (MarketScan Database, 2001–2010) and the Intercontinental Marketing Services LifeLink Database (LifeLink Database, 2001–2008)–were pooled for analyses. Both databases comprise medical (i.e., facility and professional service) and outpatient pharmacy claims from a large number of participating private health plans, and each contains claims data for 15 million persons annually.

Data available for each facility and professional-service claim include date and place of service, diagnoses, procedures performed/services rendered, and quantity of services (professional-service claims). Data available for each retail pharmacy claim include the drug dispensed, dispensing date, quantity dispensed, and number of days supplied. All claims also include paid (i.e., reimbursed) amounts. Selected demographic and eligibility information is available for persons in both databases. All data can be arrayed to provide a detailed chronology of all medical and pharmacy services used by each plan member over time.

The study databases were de-identified prior to their release to study investigators, as set forth in the corresponding Data Use Agreements. The study databases have been evaluated and certified by independent third parties to be in compliance with the Health Insurance Portability and Accountability Act (HIPAA) of 1996 statistical de-identification standards and to satisfy the conditions set forth in Sections 164.514 (a)-(b) 1ii of the HIPAA Privacy Rule regarding the determination and documentation of statistically de-identified data**.** Use of the study databases for health services research was determined–via independent third parties–to be fully compliant with the HIPAA Privacy Rule and federal guidance on Public Welfare and the Protection of Human Subjects [29].

### Study population

The study population comprised all patients who initiated ≥1 course of myelosuppressive chemotherapy for a solid tumor or non-Hodgkin’s lymphoma (NHL) and who received daily filgrastim prophylaxis during ≥1 cycle. All cycles in which patients received daily filgrastim prophylaxis–irrespective of duration–were pooled for analyses.

#### Cancer chemotherapy patients

All patients, aged ≥18 years, who began ≥1 new course of myelosuppressive chemotherapy were identified; the enrollment window was July 1, 2001 through June 30, 2010 for patients in the MarketScan Database and July 1, 2001 through June 30, 2008 for patients in the LifeLink Database. Receipt of chemotherapy was ascertained based on the presence of ≥1 paid medical claim for a chemotherapy drug or administration thereof (identified using Healthcare Common Procedure Coding System [HCPCS], International Classification of Disease, Ninth Revision, Clinical Modification [ICD-9-CM], and Health Care Financing Administration Uniform Bill-92 [UB-92] revenue codes).

Patients were considered to have initiated a new course of chemotherapy if there was a chemotherapy claim during the study period that was preceded by a period ≥60 days without any other claims for chemotherapy. Only patients who had evidence of a primary solid tumor or NHL (based on ≥2 medical claims [≥7 days apart] with a qualifying 3-digit ICD-9-CM diagnosis code during the period beginning 30 days prior to the index date and ending 30 days thereafter) were selected.

#### Chemotherapy courses, cycles, and regimens

For each cancer chemotherapy patient, each unique cycle within each course of chemotherapy was identified. The first chemotherapy cycle (of the first course) was defined as beginning with the date of initiation of chemotherapy and ending with the first service date for the next administration of chemotherapy administration (as evidenced by a medical claim with a corresponding HCPCS, ICD-9-CM, or UB-92 code) occurring at least 12 days-but no more than 59 days-after the date of initiation of chemotherapy. If a second chemotherapy cycle did not commence prior to day 60, or if there was evidence of receipt of radiation therapy (based on medical claims with relevant HCPCS, ICD-9-CM, or UB-92 codes) during this period, both the first cycle of chemotherapy and the course of chemotherapy were considered to have been completed 30 days following the beginning of the cycle or on the day prior to initiation of radiation therapy, whichever occurred first. The second and all subsequent cycles of chemotherapy, as well as subsequent courses of chemotherapy–if any–during the period of interest, were similarly defined. A maximum of 8 cycles per course were considered. For patients with multiple courses of chemotherapy, all qualifying courses were considered.

Chemotherapy regimens were ascertained based on a review of all HCPCS Level II codes for parenterally administered antineoplastic agents on medical claims with service dates within 6 days of the start of each cycle of chemotherapy. Regimens were categorized on a cycle-specific basis according to the number of agents administered that are considered to be myelotoxic (list available from authors upon request).

#### Daily filgrastim prophylaxis

Cancer chemotherapy patients who received daily filgrastim prophylaxis during ≥1 cycle of chemotherapy were selected for inclusion in the study population. Administration of daily filgrastim on or before day 5 of a given cycle was considered to represent prophylaxis [14,22,23]. Receipt of daily filgrastim was identified based on medical claims (J1440, J1441) with relevant codes from the HCPCS system.

Duration of daily filgrastim prophylaxis during the patient-cycle was characterized based on temporal patterns of administration; end of prophylaxis was defined as a gap ≥3 days in its use, outpatient administration of IV antimicrobial therapy (an indicator for possible CINC), or hospitalization for CINC. Duration of prophylaxis was characterized as 1–3, 4–6, or ≥7 days.

#### Exclusion criteria

Patient-cycles were excluded from the analytic file if the following occurred: (1) evidence of ≥2 primary cancers (solid or blood) within (i.e., +/-) 30 days of chemotherapy initiation; (2) any gaps in health benefits during the 6-month ("pretreatment") period prior to chemotherapy initiation; (3) evidence of hematopoietic stem cell or bone marrow transplantation prior to or during receipt of chemotherapy; (4) evidence of chemotherapy based only on medical claims for administration of the drugs (HCPCS codes identifying the specific chemotherapy agents were not available, and thus the regimen and level of myelosuppression could not be determined); (5) pharmacy claims for myelotoxic chemotherapy (only drug dispense dates are available on pharmacy claims, and because pharmacy/medical claims cannot be definitively linked, precise dates of chemotherapy administration–needed to characterize the course and cycles–could not be ascertained); (6) pharmacy claims for daily filgrastim (precise dates of administration could not be ascertained, for reasons stated above); (7) evidence of receipt of sargramostim (J2820) or pegfilgrastim (C9119, S0135, J2505) during the first 5 days of the cycle (i.e., as prophylaxis).

### Neutropenic complications requiring inpatient care

CINC was identified based on inpatient admissions with a diagnosis (principal or secondary) of neutropenia (ICD-9-CM 288.0), fever (780.6), or infection (list available upon request). Admissions were identified on a cycle-specific basis using acute-care facility inpatient claims with admission dates anytime between day 6 and the last day of the chemotherapy cycle. Episodes of CINC treated exclusively on an outpatient basis were not considered. Because CINC that occurred on the day of the last dose of prophylaxis or the following day could have resulted in an artificial truncation of a planned longer course of prophylaxis–and thus could exaggerate the risk of CINC with shorter courses–analyses were conducted alternatively with and without inclusion of these patient cycles.

Consequences of CINC requiring inpatient care were characterized in terms of in-hospital mortality, total hospital LOS (during the cycle), and total CINC-related healthcare expenditures (i.e., from hospital discharge to end of cycle). Total CINC-related healthcare expenditures included those for the initial hospitalization as well as care provided post-discharge on an outpatient basis; outpatient expenditures comprised encounters with a diagnosis of neutropenia, fever, or infection, as well as use/prescriptions for CSF agents (i.e., as treatment) and antimicrobial therapy. Expenditures were estimated based on total paid amounts on corresponding claims. Mortality was characterized using data only from the MarketScan Database as such information was not available in the LifeLink Database.

### Patient characteristics

Patient characteristics included: age; gender; presence of selected chronic comorbidities (cardiovascular disease, diabetes, liver disease, renal disease); history of blood disorders (anemia, neutropenia, other), infection, hospitalization (all-cause and CINC-related, respectively), chemotherapy, and radiation therapy; pre-chemotherapy healthcare expenditures; type of cancer, presence of metastases; cycle number and minimum length of prior cycles; chemotherapy regimen; receipt of antimicrobial prophylaxis in the cycle of interest; and year of chemotherapy initiation. Only observed data were utilized in defining patient characteristics.

Age was assessed as of the first day of the first cycle of chemotherapy in the course. Chronic comorbidities and history of blood disorders, infections, hospitalization, chemotherapy, radiation therapy, presence of metastases, and healthcare expenditures were assessed from the beginning of the 6-month pretreatment period through the first day of the corresponding cycle of chemotherapy. Selected variables (i.e., anemia, neutropenia, other blood disorders, infections) were alternatively evaluated during the chemotherapy course (up to the beginning of the cycle of interest). Metastases (bone vs. other site) and chronic comorbidities were identified on the basis of ≥1 diagnosis codes on inpatient claims, ≥2 diagnosis codes on outpatient claims (excluding those for laboratory services) on different days, ≥1 procedure codes, and ≥1 drug codes, as appropriate. Blood disorders and infections were identified on the basis of ≥1 diagnosis codes (on inpatient and/or outpatient claims) and ≥1 drug codes, as appropriate. Prophylactic use of antimicrobial agents was ascertained based on a medical claim for administration of drug from cycle day 1 to cycle day 5, or a pharmacy claim for a filled prescription from cycle day -3 to cycle day 5, with a corresponding drug code.

### Statistical analyses

Incidence of CINC was evaluated for each patient-cycle in which daily filgrastim was administered prophylactically, and was estimated by same-cycle duration of prophylaxis in an unadjusted and adjusted context. For the latter, a generalized estimating equation (GEE) with a binomial distribution, logistic link function, and exchangeable correlation structure was employed. The GEE method accounts for correlation among repeated measures for the same subject (in this instance, across cycles), while controlling for both fixed characteristics (e.g., gender) and time-dependent covariates (e.g., first versus subsequent cycles). All observed patient characteristics were entered into, and retained in, the multivariate model. Subgroup and sensitivity analyses focusing on the first cycle only, employing a narrow definition of CINC (which was identified using the diagnosis code for neutropenia only but otherwise the same algorithm for the broad definition as described above), and focusing on alternative tumor types separately (i.e., breast cancer, lung cancer, colorectal cancer, and NHL) also were conducted.

In-hospital mortality, hospital LOS, and CINC-related healthcare expenditures among patients who developed CINC were descriptively evaluated by duration of daily filgrastim prophylaxis. Confidence intervals (95%) for in-hospital mortality were computed using the Wilson score interval; confidence intervals for LOS and economic costs were computed using nonparametric bootstrapping (percentile method) from the study population (1,000 replicates with replacement). Confidence intervals for in-hospital mortality, hospital LOS, and healthcare expenditures were estimated assuming independence between observations. Only observed data were used in defining study variables; patients who developed CINC but were missing data on study measures–because such data either were not recorded on claims or were not provided by some health plans–were excluded from corresponding analyses.

## Results

**Figure 1:**
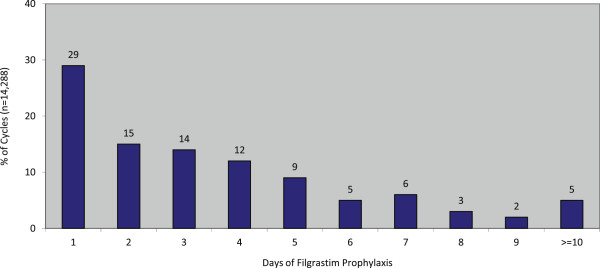
Days of filgrastim prophylaxis.

A total of 135,921 adult patients initiated a new course of chemotherapy for a solid tumor or NHL during the period of interest and met all other eligibility criteria. Among these patients, a total of 5,477 received daily filgrastim as prophylaxis during ≥1 cycle of chemotherapy and thus were included in the study population; these patients contributed a total of 14,288 daily filgrastim prophylaxis patient-cycles to the analytic file. Filgrastim was administered for 1–3 days in 58% of cycles (n = 8,371), 4–6 days in 26% of cycles (n = 3,691), and ≥7days in 16% of cycles (n = 2,226) (Figure 1).

Mean (SD) age of patients was 54 (13) years for 1–3 days of filgrastim prophylaxis, 57 (14) years for 4–6 days of prophylaxis, and 56 (13) years for ≥7 days of prophylaxis (Table 1). Breast cancer was the most common tumor type (48% for 1–3 days, 50% for 4–6 days, 49% for ≥7 days), followed by NHL (10%, 13%, and 13%, respectively), and colorectal cancer (16%, 10%, and 13%, respectively). Metastatic disease was present in 38% of patients receiving 1–3 days of prophylaxis, 32% receiving 4–6 days, and 35% receiving ≥7 days. Antimicrobial agents—principally oral (~90%)—were concurrently used as prophylaxis in 6.7% of patients receiving 1–3 days of filgrastim prophylaxis, 7.5% receiving 4–6 days, and 7.5% receiving ≥7 days.

**Table 1:** **Patient**, **cancer, and treatment characteristics**, **by duration of filgrastim prophylaxis**

	**Study population**, **by days of filgrastim prophylaxis**		
	**1-3 (n = 8,371)**	**4-6 (n = 3,691)**	**> = 7 (n = 2,226)**
**Patient**			
**Age (years), mean (SD)**	54.1 (12.5)	57.4 (13.7)	59.2 (12.9)
**Men, %**	23.5	23.1	21.7
**Chronic comorbidities, %**			
Cardiovascular disease	1.6	2.0	1.9
Diabetes	8.2	10.2	7.3
Liver disease	1.0	0.9	0.5
Renal disease	0.9	1.0	1.4
**History of other Conditions/Events Prior to Chemotherapy Course, %**			
Anemia	15.2	12.3	14.5
Neutropenia	6.1	5.5	5.7
Other blood disorders	2.2	2.0	2.4
Infection	24.6	33.1	35.8
History of hospitalization for any reason, %	50.6	49.3	49.7
History of CINC-related hospitalization, %	6.6	9.1	10.4
History of chemotherapy, %	9.0	6.4	6.9
History of radiation therapy, %	6.5	4.3	4.0
**Pre-Chemotherapy Expenditures ($), Mean ± SD**	34,736 (43,618)	33,913 (42,458)	34,480 (43,828)
**History of conditions/events in prior cycles of same chemotherapy course, %**			
Anemia	18.5	18.5	19.6
Neutropenia	38.0	45.4	44.9
Other blood disorders	1.5	1.5	2.2
Infection	31.9	34.2	34.3
**Cancer**			
**Type, %**			
Female breast	48.2	49.6	49.6
Non-Hodgkin’s Lymphoma	9.9	12.8	23.3
Trachea, bronchus, lung	7.5	9.5	8.1
Prostate	0.6	0.5	0.1
Colon/Rectum	15.9	9.8	4.6
Other	17.9	17.9	14.2
**Presence of metastases, %**			
Bone	2.5	1.5	1.8
Other Site	35.1	30.3	29.6
**Chemotherapy**			
**Cycle number/min. length of prior cycles in same chemotherapy course, %**			
1	12.6	13.1	16.3
2/12-19	5.9	4.3	4.8
2/20-26	8.3	10.8	12.1
2/27+	2.1	3.0	2.7
> = 3/12-19	41.5	31.8	21.1
> = 3/20-26	27.8	35.3	40.5
> = 3/27+	1.7	1.8	2.5
**Number of Myelosuppressive Drugs, %**			
1	24.3	20.0	14.5
2	48.0	48.2	51.3
3	20.6	25.4	31.3
> = 4	7.1	6.4	3.0
**Receipt of Antimicrobial Prophylaxis, %**	6.7	7.5	7.5
**Year of Chemotherapy, %**			
2001-2003	19.2	19.0	26.9
2004-2006	39.2	34.2	34.2
2007-2010	41.7	46.8	38.9

**Figure 2:**
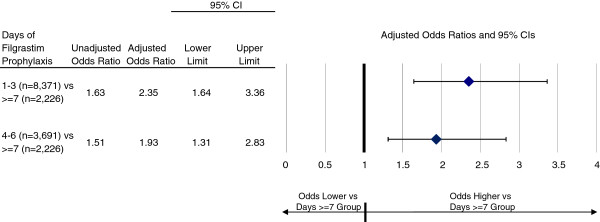
Adjusted odds ratios for chemotherapy-induced neutropenic complications requiring inpatient care, by duration of filgrastim prophylaxis*. *Results adjusted for patient, cancer, and chemotherapy characteristics.

Crude risk of CINC during a cycle of chemotherapy was 2.9% with 1–3 days of filgrastim prophylaxis, 2.7% with 4–6 days, and 1.8% with ≥7 days. In adjusted analyses, the odds of CINC were 2.4 (95% CI: 1.6-3.4) and 1.9 (1.3-2.8) times higher with 1–3 and 4–6 days of filgrastim prophylaxis, respectively, versus ≥7 days (referent group) (Figure 2). Among the subgroup of patients who developed CINC requiring inpatient care (n = 382), mean hospital LOS was 7.4 (6.4-8.3) days with 1–3 days of prophylaxis (n = 243), 7.1 (5.7-8.5) days with 4–6 days of prophylaxis (n = 99), and 6.5 (4.9-8.0) with ≥7 days of prophylaxis (n = 40) (Table 2). Among the subgroup of patients who developed CINC and for whom healthcare expenditures were available (n = 358), mean total CINC-related healthcare expenditures were $18,912 (14,570-23,581) with 1–3 days of prophylaxis (n = 225), $14,907 (11,155-19,728) with 4–6 days (n = 94), and $13,165 (9,595-17,144) with ≥7 days (n = 39). Among the subgroup of patients who developed CINC and for whom discharge status was available (n = 228), in-hospital mortality was 8.4% (4.6-14.8) with 1–3 days of prophylaxis (n-119), 4.0% (1.4-11.1) with 4–6 days (n = 75), and 0% (0–10.2) with ≥7 days (n = 34).

**Table 2:** **Unadjusted risk of inpatient mortality**, **length of stay in hospital**, **and healthcare expenditures for chemotherapy**- **induced neutropenic complications requiring inpatient care**, **by duration of filgrastim prophylaxis**

	**Days of Filgrastim Prophylaxis**		
	**1-3**	**4-6**	**> = 7**
Mortality, % (95% CI)	8.4 (4.6-14.8)	4.0 (1.4-11.1)	0.0 (0.0-10.2)
	(n = 119)	(n = 75)	(n = 34)
LOS (days), mean (95% CI)	7.4 (6.4-8.3)	7.1 (5.7-8.5)	6.5 (4.9-8.0)
	(n = 243)	(n = 99)	(n = 40)
Expenditures, mean (95% CI)			
Inpatient	$18,509 (14,195-23,145)	$14,527 (10,758-19,243)	$12,544 (9,063-16,315)
Outpatient	$226 (126–350)	$356 (148–630)	$581 (145–1,180)
Pharmacy	$177 (68–329)	$24 (13–39)	$41 (10–88)
Total	$18,912 (14,570-23,581)	$14,907 (11,155-19,728)	$13,165 (9,595-17,144)
	(n = 225)	(n = 94)	(n = 39)

*n’s indicate the number of patient-cycles considered in analyses of study measures; among patients who developed CINC (n = 382), those with missing data on study measures- because such data were not recorded on claims or were not provided by some health plans- were excluded from corresponding analyses.

**Only patients with paid amounts > $0 were considered in these analyses.

In subgroup analyses focusing on the first cycle only, crude risks of CINC were 7.0% with 1–3 days of filgrastim prophylaxis (n = 1054), 5.0% with 4–6 days (n = 482), and 5.2% with ≥7 days (n = 363); in adjusted analyses, odds of CINC were 1.6 (0.9-2.8) and 1.1 (0.6-2.1) times higher with 1–3 and 4–6 days (versus ≥7 days) of filgrastim prophylaxis in cycle 1 (Table 3). In analyses employing the narrow definition for CINC, crude risks of CINC were 1.6% with 1–3 days of filgrastim prophylaxis, 1.8% with 4–6 days, and 1.3% with ≥7 days; in adjusted analyses, odds of CINC were 1.6 (1.1-2.5) and 1.7 (1.1-2.7) times higher with 1–3 and 4–6 days of filgrastim prophylaxis, respectively, versus ≥7 days. In tumor-specific analyses, adjusted odds of CINC with 1–3 and 4–6 days of filgrastim prophylaxis (vs. ≥7 days) were: 1.8 (1.0-3.0) and 1.9 (1.1-3.4) for breast cancer; 20.2 (1.9-212.4) and 14.5 (1.3-158.7) for lung cancer; 1.9 (0.2-16.7) and 2.5 (0.3-23.5) for colorectal cancer; and 2.1 (1.2-3.6) and 1.8 (1.0-3.2) for NHL. We note that not all observed differences were statistically significant in unadjusted subgroup and secondary analyses, presumably due to the lack of adjustment for systematic differences in patient characteristics between prophylaxis subgroups.

**Table 3:** **Adjusted odds ratios for chemotherapy**- **induced neutropenic complications requiring inpatient care in subgroup and secondary analyses**

				**Unadjusted**	Adjusted*
	**No. of cycles**	**No. of events**	**%**	**Odds ratio**	**Odds ratio**
**Narrow definition of CINC**					
**Days of Filgrastim Prophylaxis**					
1-3	8,371	130	1.6%	1.15 (0.77,1.72)	1.60 (1.05,2.45)
4-6	3,691	67	1.8%	1.35 (0.88,2.09)	1.74 (1.11,2.72)
> = 7	2,226	30	1.3%	–	–
**Broad definition of CINC**					
**First cycle only**					
**Days of Filgrastim Prophylaxis**					
1-3	1,054	74	7.0%	1.37 (0.81, 2.30)	1.56 (0.89, 2.74)
4-6	482	24	5.0%	0.95 (0.51, 1.76)	1.11 (0.58, 2.12)
> = 7	363	19	5.2%	–	–
**Breast cancer**					
**Days of Filgrastim Prophylaxis**					
1-3	4,038	94	2.3%	1.44 (0.86, 2.39)	1.75 (1.02, 3.00)
4-6	1,829	49	2.7%	1.66 (0.96, 2.86)	1.92 (1.09, 3.38)
> = 7	1,103	18	1.6%	–	–
**Lung cancer**					
**Days of Filgrastim Prophylaxis**					
1-3	630	44	7.0%	13.52 (1.85, 98.79)	20.20 (1.92, 212.36)
4-6	349	15	4.3%	8.08 (1.06, 61.70)	14.54 (1.33, 158.65)
> = 7	181	1	0.6%	–	–
**Colorectal cancer**					
**Days of Filgrastim Prophylaxis**					
1-3	1,327	21	1.6%	1.64 (0.22, 12.32)	1.88 (0.21, 16.67)
4-6	360	9	2.5%	2.62 (0.33, 20.89)	2.54 (0.27, 23.54)
>=7	103	1	1.0%	–	–
**NHL**					
**Days of Filgrastim Prophylaxis**					
1-3	830	63	7.6%	1.86 (1.13, 3.05)	2.06 (1.19, 3.55)
4-6	474	28	5.9%	1.42 (0.80, 2.52)	1.76 (0.95, 3.23)
> = 7	519	22	4.2%	–	–

*Results adjusted for patient, cancer, and treatment characteristic listed in Table 2.

## Discussion

In the largest retrospective study of daily filgrastim use in US clinical practice, we found that the large majority of patients undergoing cancer chemotherapy who are administered prophylaxis with daily filgrastim receive considerably fewer days of administration than subjects in clinical trials of daily filgrastim prophylaxis. In our study population, 95% of patients received fewer than 10 days of filgrastim prophylaxis and 58% received only 1–3 days, versus the typical 10–11 days required for neutrophil recovery in the pivotal clinical trials [17-19]. This finding is largely consistent with observations from other retrospective clinical practice studies [14-16,22,23]. We also found that patients who receive shorter courses of daily filgrastim prophylaxis have a substantially higher risk of CINC requiring hospitalization. Among patients in our study population who received 1–3 or 4–6 days of prophylaxis, the adjusted odds of CINC were 2.4 and 1.9 times, respectively, higher than those receiving ≥7 days. In addition, our study results suggest that, among the subgroup of patients who develop CINC despite prophylaxis, the consequences of this condition may be worse among those who receive fewer administrations of daily filgrastim prophylaxis. This last set of results should be interpreted with caution, however, as the number of patients who developed CINC despite prophylaxis was, in absolute terms, small.

Over the past decade, the frequency of use of alternative (often, more myelosuppressive) chemotherapy regimens in US clinical practice has changed considerably, in large part due to better chemotherapy agents, combinations of agents, and dosing schedules [25-27]. Because these regimens are typically associated with higher levels of myelosuppression, use of supportive care–including CSF prophylaxis–also has increased [27]. While pegfilgrastim—a longer-acting version of filgrastim that requires only a single dose administered subcutaneously once per chemotherapy cycle—is now (by far) the most commonly used CSF prophylactic agent in US clinical practice, filgrastim accounts for a small but important segment of the prophylactic market [14,16]. In the present study, 60,600 (45%) of the 135,921 adult cancer chemotherapy study subjects received CSF prophylaxis in ≥1cycle during their chemotherapy course. Among the subgroup who received CSF prophylaxis, 91% received pegfilgrastim in ≥1 cycle and 9% received filgrastim. Notwithstanding these temporal changes in chemotherapy regimens and supportive care, however, our findings regarding the frequent use of shorter courses of filgrastim prophylaxis and the associated consequences–based on a study population of over 5,000 patients and nearly 15,000 patient-cycles–are largely consistent with those reported previously. In the 2006 study by Weycker and colleagues–which included 598 breast cancer, lung cancer, and NHL patients, and employed data from 1998-2002– for example, mean duration of prophylaxis ranged from 4.3 to 6.5 days across tumor types, while in the Morrison study–which included 1,451 cancer patients and employed data from 2001-2003– mean duration of prophylaxis ranged from 3.7 to 6.0 days across calendar years and cycles of use [15,23]. In the present study, mean (SD) duration of prophylaxis on an overall basis was 3.6 (2.9) days. Moreover, in the aforementioned 2011 study by Weycker et al., odds of CINC were reported to be 1.5 times higher for patients receiving <7 versus ≥7 days of filgrastim prophylaxis, while the corresponding odds ratio in this study was estimated to be 2.2 (95% CI 1.6-3.1) [14]. We note that odds ratios for CINC reported above were largely comparable when limiting attention to the most recent four-year period (2007–2010): 2.5 (1.5-4.3) with 1–3 versus ≥7 days of filgrastim prophylaxis and 2.2 (1.2-3.9) with 4–6 versus ≥7 days of filgrastim prophylaxis. Study results also were robust to alternative specifications of the multivariate model and when excluding observations for which hospitalizations occurred on the day of the last dose of daily filgrastim prophylaxis or the following day.

We expected that systematic differences in the prevalence of risk factors (e.g., history of neutropenia, higher doses of chemotherapy agents, metastases to bone, poor performance status) for CINC would occur according to the duration of prophylaxis (1–3, 4–6, vs. ≥7 days). We thus used techniques of multivariate regression to adjust for such risk factors–to the extent possible–in analyzing the relationship between duration of daily filgrastim prophylaxis and outcomes of interest. Given the limitations of the claims-based databases we used, however, we were forced to use proxies for certain established risk factors. For example, a proxy measure based on pre-chemotherapy healthcare expenditures was employed for performance status, which in prior research has been shown to be correlated with health status in other patient populations [30]. Moreover, because information was not available for some clinically important parameters (e.g., ANC), the possibility exists that the study groups differed in terms of unobserved characteristics that predispose to CINC.

We believe that our approach to controlling for such systematic differences was comprehensive given available data, and that the above-noted biases–if present–would confer a conservative bias to analyses. For example, if the risk of FN is higher among patients receiving higher doses of chemotherapy (which is unobservable in the study database), and these patients are more likely to receive longer durations of prophylaxis, then the estimated difference in risk between patients receiving longer versus shorter courses of prophylaxis will be smaller than the true or actual difference. That our adjustment procedures were at least to some extent successful is suggested by the greater odds ratios for CINC in the adjusted versus unadjusted analyses, but uncertainty as to the adequacy of adjustment for confounding risk factors is one of the major limitations of our study.

In those instances where code-based operational algorithms were used to identify risk factors of interest (e.g., using ICD-9-CM code 288.0 for neutropenia, rather than ANC) errors of omission/commission in medical coding may have impacted the accuracy of adjustment. We do not believe, however, that any such differences or limitations were for the most part systematic in nature. However, patients who have a history of CINC – and thus may be more likely to receive longer durations of prophylaxis – may be more likely to have "neutropenia" designated as a secondary (or even primary) diagnosis on future encounters, all else equal.

There is no ICD-9-CM diagnosis code for CINC (i.e., neutropenia-related fever or infection), and thus codes for neutropenia, fever, and infection were employed to identify hospitalizations assumed to be related to neutropenic complications. Patients are typically not given chemotherapy when they are neutropenic or have active infection. The timing of fever and infection after chemotherapy increases the likelihood that such outcomes are related to receipt of chemotherapy. Codes for neutropenia, fever (surrogate for active infection), and infection thus are all likely to be related to episodes if seen within a defined exposure period after receiving chemotherapy. While the sensitivity of this algorithm for identifying neutropenic complications is undoubtedly higher than that of an algorithm using only the ICD-9-CM code for neutropenia, its specificity and positive predictive value are unknown.

Other miscellaneous limitations deserve a brief mention. CINC requiring outpatient care were not considered in our analyses due to the small total number of events (n = 60). Patients with evidence of receipt of daily filgrastim via outpatient pharmacy (3% of total) were excluded because we could not ascertain the precise dates of use. Expenditures were not adjusted to current dollars since use of a general price index may yield spurious findings when applied to specific patients in specific health plans who consumed specific healthcare services and since the distribution of study groups by calendar year was comparable. Hospital discharge disposition was available only in the MarketScan Database and information concerning LOS or paid amounts was missing for some hospitalizations. Moreover, we note that a disproportionately high percentage of patients receiving 1–3 days of filgrastim prophylaxis (vs. those receiving 4–6 or ≥7 days) who developed CINC requiring inpatient care did not have information available on discharge disposition, and thus the inpatient mortality findings must be viewed with caution. Although patients initiating "delayed" use of daily filgrastim (e.g., after day 5 of the chemotherapy cycle) have been included in other published evaluations, they were not considered in our analyses as such patients may have received daily filgrastim for the treatment of CINC (e.g., severe neutropenia) as opposed to prophylaxis against CINC [15,16]. Finally, we note that it cannot be determined from outpatient pharmacy claims data whether drugs dispensed were actually taken, when they were taken, or how much was taken. For this reason, our characterization of antimicrobial prophylaxis use (principally oral) may be upwardly biased and differences in actual use across prophylaxis subgroups may confound the results of analyses. We also note, however, that the percentage of patients with filled prescriptions for antimicrobials was relatively low (and comparable) across filgrastim prophylaxis subgroups.

## Conclusion

In conclusion, we found that among patients receiving myelosuppressive chemotherapy, shorter courses of daily filgrastim prophylaxis are associated with increased risk of CINC. We also found that when CINC develops despite daily filgrastim prophylaxis, the outcomes thereof may be poorer in those receiving shorter courses of prophylaxis. Additional research is needed to explore these relationships among individual tumor types and chemotherapy regimens.

## Competing interests

Funding for this research was provided by Amgen Inc. to Policy Analysis Inc. (PAI). Derek Weycker, John Edelsberg, and Alex Kartashov are employed by Policy Analysis Inc. (PAI). Rich Barron and Jason Legg are employed by Amgen Inc. Andrew Glass is employed by the Center for Health Research, Kaiser Permanente Northwest, and received an honorarium for this research from Amgen Inc.

## Authors’ contributions

Authorship was designated based on the guidelines promulgated by the International Committee of Medical Journal Editors (2004). All persons who meet criteria for authorship are listed as authors on the title page. The contribution of each of these individuals to this study–by task–is as follows: conception and supervision (Barron, Weycker), development of design (Barron, Edelsberg, Glass, Legg, Weycker), conduct of analyses (Kartashov, Weycker), interpretation of results (all authors), preparation of manuscript (Edelsberg, Weycker), and review of manuscript (all authors). All authors have read and approved the final version of the manuscript. The study sponsor reviewed the study research plan and study manuscript; data management, processing, and analyses were conducted by Policy Analysis Inc. (PAI), and all final analytic decisions were made by study investigators.

## Pre-publication history

The pre-publication history for this paper can be accessed here:

http://www.biomedcentral.com/1472-6963/14/189/prepub

## Declaration of financial/other relationships

Derek Weycker, John Edelsberg, and Alex Kartashov are employed by Policy Analysis Inc. (PAI). Rich Barron and Jason Legg are employed by Amgen Inc. Andrew Glass is employed by the Center for Health Research, Kaiser Permanente Northwest, and received an honorarium for this research from Amgen Inc.
